# Real-World Ethical Dilemmas in Laboratory Safety for Microbiology Under-Resourced and Outreach Teaching

**DOI:** 10.3389/fmicb.2021.589569

**Published:** 2021-04-07

**Authors:** Beverly L. Smith-Keiling

**Affiliations:** ^1^Department of Biochemistry, Molecular Biology, and Biophysics, University of Minnesota Medical School and College of Biological Sciences, Minneapolis, MN, United States; ^2^Division of Epidemiology & Community Health, University of Minnesota School of Public Health, Minneapolis, MN, United States

**Keywords:** ethics, laboratory, safety, policy, outbreak, risk, curriculum, equity

## Abstract

With modernization of safety standards for microbiology outreach teaching laboratories, ethical challenges arise in teaching microbiology for the public good without short-changing students in under-resourced situations, or when institutional support is subpar. Still, educators want students to engage using applied skills for inquiry, research-based microbial learning activities – safely. Following several United States microbial outbreaks, federal investigation traced sources back to teaching laboratories. Policy discussions ensued. The American Society for Microbiology (ASM) Task Force provides recommended but not mandated guidelines; however, guidelines are not amenable by all. Here, a real-world, ethical scenario of a university-level outreach microbiology laboratory course hosted at several locations provides context for under-resourced challenges in safety compliance. In this example of biomedical and public health ethical considerations, upper administration puts the onus on instructors to assure safe labs for their students and the general public. Temporarily hired instructors without curriculum or sufficient institutional support are put in precarious positions with often egregious practices to get the job done. This scenario is examined with different public health ethical frameworks and principles: non-maleficence, beneficence, health maximization, efficiency of policy regulations, respect for institutional and instructor autonomy, justice, and proportionality balancing stakeholder concerns. Sample curricular strategies are employed to mitigate these challenges. Taking a utilitarianism framework of the greatest good for the most benefit, this paper advocates for social justice supporting access to education as a moral duty. Administrations should ensure instructors are supported sufficiently to provide safe, authentic learning experiences. Solutions for under-resourced outreach teaching are needed for public trust.

## Introduction

Teaching microbiology laboratory courses safely has new meaning and ethical challenges. Even before modern life-altering pandemics begin changing worldviews, raised awareness is needed of ethical safety challenges faced in under-resourced science teaching laboratories. Change away from “normal science” practice creates tensions. Reasoning helps “puzzle-solve” through crisis (Kuhn, 1962). Exploring ethical dilemmas helps balance competing needs such as increasing stringency of safety in resource-limited settings without limiting learning, sustaining equitable educational opportunities, and negotiating administrative priorities. Every accident or near miss, whether biological or chemical, teaches lessons reminding that safety is integral to science. Worst-case scenario emerging pathogen pandemic planning is attentive to history, changing paradigms in biosafety, social justice, and ethical lenses to mitigate disease. All are trademarks applying public health perspectives (Mack et al., 2007) also necessary in the small-scale educational setting.

Comprehensive, updated biosafety sources (Wooley and Byers, 2017) include specific recommendations addressing the special environment of the college-level teaching laboratory and recognizing burdens and liabilities of instructors from under-resourced settings (Woolverton and Woolverton, 2017). Additional resources and CDC biosafety training modules^1^ (Table 1) can assist institutional decision-making capabilities to maintain safe standards, even when staff may lack legal protection when some institutions avoid compliance. Generally, biosafety officers assist instructors to assure safe student instruction environments. Without institutional support and oversight, sometimes the instructor alone makes the decision to use practices beyond biosafety level (BSL)1 criteria, conducted on a standard laboratory table with minimal personal protective equipment (PPE), e.g., optional gloves and eye protection. Some practices, e.g., discouraged isolation from environmental sources, could isolate BSL2 organisms and pose infectious risk. Even well-equipped laboratories working within established laboratory safety practices have risk (Hayden, 2011) as seen in several multi-state outbreaks^2^,^3^,^4^ of a pathogenic strain of *Salmonella* Typhimurium originating in clinical and teaching laboratories.

**Table 1:** Sample University X under-resourced teaching laboratory scenario.

Real-world scenarios	Challenge	Under-resourced responses, solutions, and persistent remaining challenges
***Under-resourced example***		
University X, a United States-based, originally brick-and-mortar institution teaches university courses at globally-located sites in host countries and online. Serves a diversity of underrepresented and lower SES students, providing a valuable education and next step. Provides STEM core courses for credentials.	Unique settings present challenges for different science laboratory courses – particularly microbiology. Mission to keep tuition low creates culture of scarcity. Need to assure courses are sustained for STEM pipeline discoveries, innovations, health and economic growth with adequate numbers of research and public health professionals.	Unique solutions to daily challenges are sought. Some solutions employed are not within guidelines. Less costly supervisory roles lack safety-trained leadership. Mandates and guidelines may be ignored if not enforceable, yet puts greater moral burden and culpability on instructors.
***Failure to meet compliance***		
United States Occupational Safety and Health Act of 1970 (OSH Act, 2011). Institution provided laboratory courses practices common to most institutions until 1990 OSHA Laboratory Standard regulations for safety education requires protection for employees from hazards causing serious harm. Regulations specify Chemical Hygiene Officer, written Chemical Hygiene Plans, and generally a biosafety officer. University X neither kept up, nor meets OSHA regulations; faculty and OSHA-trained students recognize breaches; faculty risk-takers have higher tolerance for poor compliance; students grateful for education are less likely to report complaints. Administration lacking trained guidance makes top-down, unilateral decisions directly impacting educational safety standards: cuts online microbiology laboratory courses, allows other coursework to continue without safety oversight, and remains in violation of biomedical ethical standards in many host countries abroad for non-compliant storage, transport, access, and use of chemical and biological materials. Institutional policies to inform faculty and provide training support lack leadership. Those with more knowledge hold greater responsibility and culpability if an accidental exposure and/or outbreak occurred without power to address the issues. Internal and external whistleblowing is a response as a consequence of a failed action.	Institutional non-compliance. Inability to keep up with safety changes without allocated resources and leadership. Exempted or non-compliant from mandates e.g., education contracted on military bases with exemptions, online education, or by negligence. Administrative decision-making by Dean and Academic Coordinators under the approval of the top administration is flawed without consultation with experts and solutions. Responsibility, culpability, and autonomy are complex.	Administration seeks steps to compliance: - OSHA Occupational Safety and Health Administration (2011). Administration hires support staff. Provide faculty training and safety officer assistance. Accessible resources: - updated (Wooley and Byers, 2017) built on prior safety guidelines (Fleming and Hunt, 2006). - Special teaching laboratory environment (Woolverton and Woolverton, 2017). - ASM resources.^8^ - International recommendations World Health Organization (WHO, 2004). - Online resources (Barber and Stark, 2015). - Biosafety Level (BSL) criteria for risk assessment learned through Center for Disease Control (CDC) training.^1^ - National Research Council guidelines for chemicals (National Research Council, 2011, 2014). Policy change can support faculty with safe compliant, low-cost, curricula meeting educational competency needs and retaining courses.
***Under-resourced settings***		
Laboratory courses are held wherever space is available. Faculty drive to each site with supplies to set up the “lab” before class (in variable settings), teach lecture and lab, break it down, clean, dispose waste to move back to a storage area or their own homes. An advertisement depicting a remote area boldly claims “*Where others see this, we see a classroom*.” Science knows no bounds. Even non-traditional microbiology learning can take the form of a traveling lab bus for access. Without safe disposal, microbial waste is sewaged, dissected formalin-preserved specimens (from Anatomy course) put in woodchippers to “hide” waste in trash.	Outreach sites struggle with additional challenges to meet guideline compliance. With no storage in shared spaces, microbial plates are put in incubation tubs labeled “do not touch” left at “room temp” in a public office space, or personal vehicle. Diverse locations lack space for storage, sample incubation, or cleaning. Without areas to autoclave waste, everything must be cleared for immediate disposal or safe transport.	Meet logistical challenges through community effort to collectively design and provide safe curricula for different outreach settings not meeting guidelines. Attempt to collectively generate ideas. One-day experiments without stored incubation. Pressure cooker instead of autoclave or bring waste to hospital.
***Under-resourced faculty preparation***		
Faculty are temporarily hired, may have limited training in microbiology, little practical skill, nor aware of ASM resources or safety guidelines. With lack of guidance, institution remains unaware of current standards. Administration puts the onus on instructors to assure safe laboratory courses with assumptions that temporarily hired instructors have basic knowledge to teach the course. Those coming from well-supported institutions struggle with low resources and do not recognize unintended consequences of stringent guidelines. Faculty relying on older traditional methods are not familiar with current guidelines. Under-resourced faculty experience isolation. Without a supported lab manual curriculum, or institutional safety officer, faculty are sole decision-makers. Sole safety-aware science instructor (microbiologist) may become the lone voice seeking institutional change. Frustration mounts when the institution lacks safety officer to support faculty or designate faculty member as biosafety leader without training or credentials. Hierarchy designates safety officers to bear the burden of liability, but places a higher burden on the instructor resulting in fears of liability, culpability, or guilt if an accident or near miss occurs. Embarrassment identifying with an institution blinded more by finances than by safety. Might not publish without compliance or fear of public exposure.	Lacking preparation and with a knowledge gap in where to find resources, guidelines, or curricula, faculty rely on their prior knowledge; older methods of isolation from environmental sources are practiced. Without stock cultures, yogurt provides the easiest source, but new temporary faculty step into the position (sometimes with less than 2 weeks to prepare) with the same challenge not knowing where to begin. Isolation keeps under-resourced faculty from finding assistance. Fear hampers reaching out *via* listservs and putting self and institution at risk of further culpability. With questionable institutional practices, not only is safety a concern but also culpability when compliance is not met. When the institution lacking safety measures does not require (or even allow) faculty publishing, then raises an ethical concern of justice.	Trained mentor or administrator provides orientation packet of resources with safety training and curricular ideas attempts to address gaps faculty face. However, with set syllabi and curricula, this results in the risk of lost autonomy in curricular decisions. Community of educators provides support.
***Under-resourced curriculum support***		
Without a curriculum, a biosafety officer or suggestions to get started, faculty relies on what is “on hand” and think outside the box. Piecemeal lab kits putting faculty in precarious positions often with egregious practices to get the job done. With sparsely allocated resources of funds, time, and staff to minimally address chemicals, PPE and biological waste disposal, the pros and cons of stringent guidelines yield different forms of harm.	Challenges meeting ASM curricular learning outcomes^6^: “Ways to properly prepare and view specimens for examination using microscopy, use pure culture and selective techniques to enrich for and isolate microorganisms and methods to identify microorganisms.”	Those with knowledge can use open-source resources indicate curricula for under-resourced needs: - ASM guidelines.^6^ - ASM resource curricula.^8^ - Course Source.^9^
***Under-resourced supplies***		
Instructors not receiving laboratory supplies, purchase materials, and transport laboratory grade chemicals in personal vehicles, sometimes across international borders. Lacking supplies, storage, safety training, etc., a laboratory kit pieced together supports several standard lab exercises. Instructors acquaint themselves with available supplies. Since no laboratory manual curriculum is available, they make due to provide a laboratory course. Faculty bringing sheep brains from farms (for Anatomy course use) were discouraged from doing (potential prion disease). Faculty working jointly in clinics bringing clinical isolates from hospital patient cases were discouraged from doing so (pathogens).	Logistics of transport, shipping, labeling, and storing supplies must be considered at every level for safety beyond home regulations. Lacking standardized training, supplies, and curricular resources, a trained safety officer or administrator could provide safe solutions. Creative solutions, such as portable eyewashes, hand wash stations, and proactive thinking attempt to meet safety needs. Autonomous faculty may use practices with risk guidelines help assist change.	Faculty reimbursement for supplies: grocery store low-budget material purchases rather than maintaining a chemical cabinet. Develop or identify safe curricula suggestions and common supplies to meet competencies. Identify standard curricula proper disposal methods or alternative solutions. Provide safe ideas for drop off facilities at hospitals or veterinary disposal for lab animals (Anatomy course).
***Risk of mandated guideline (cancelled course)***		
Paradigms changed to legally required (1990 OSHA Laboratory Standard). University X did not meet compliance as one indicator of deficiency and poor staff support. Following 2012 ASM Task Force recommended guidelines, knowledgeable faculty advocated addressing risk concerns. Raising awareness of breach in compliance internally results in some measures to attempt compliance, but not always satisfactorily: - Rather than comply, disposal of all chemicals at sites results in lower resources. - Shipping instead of driving chemicals across borders with improper transportation in personal vehicles affects laboratory exercises. - Online microbiology laboratory course cancellation and lost course opportunity. - Institutional discussions to cancel microbiology altogether considered. - Attempts to provide a safe curriculum still continue discouraged environmental isolation microbiology practices as if status quo.	Mandated-policy loopholes and recommended, non-enforceable guidelines meant administration choses only to address some safety needs. Responsiveness varies with exemptions and no watchdog. Centralized command spans large area with communication and leadership gaps. Increasing faculty concern for their safety and culpability with no trained safety officer. Raising awareness of guidelines increases faculty awareness of their risk. Kneejerk response to safety concerns results in lost course, jobs, and student access with greater impacts for low SES under-represented students.	Do not assume even legal mandates are enforced. Use bottom-up community discussions involving faculty knowledge, practices, and addition training and curricula. Suggest safely bought, stored, and transported materials. Develop curricular ideas to sustain at-risk courses and promote social justice. Even if under-resourced or low support, provide educators safety training and curricula resources for their own autonomous decision. Before course cancellation, community solutions may help.
***Potential student and staff infections***		
An introductory undergraduate University X student learned aseptic technique in microbiology laboratory training. He steadfastly isolated and identified his unknown from the “handwashing lab” paper towel environmental source. On Monday returning from a weekend of eating out at a buffet and profuse illness, he also identified his unknown as the toxin-forming *Bacillus cereus* that can cause the same symptoms, and questioned his source of illness that could have spread to his newborn or immunocompromised partner. Contrast this with an isolate originating from soil in a historically endemic area for *B. anthracis* known to persist for decades. A staff member untrained in safety cleans up plates from environmental soil samples and is unknowingly exposed. The new faculty member not knowing the risks seeks information to determine the risk of anthrax. Educator fears resurrecting the dormant endospore of the organism responsible for the Henle-Koch Postulates and Germ Theory of Disease. *Bacillus anthracis* endospores are resistant to heat, produce anthrax toxin, and are still found globally in soil and zoonosis with potential for outbreak (Koch, 1877; Evans, 1976; Meselson et al., 1994).	Without safety protocols, there is no protocol for reporting or diagnosis. Student, staff, and public at risk from laboratory practice. Commonly found *Bacillus cereus* endospores easily isolated from paper towels or tabletops can cause a toxin-induced food poisoning (Dohmae et al., 2008; Gendron et al., 2012). Dosage impacts virulence. Several *Bacillus* species found environmentally are valuable non-pathogenic surrogate models (Greenberg et al., 2010), but there is no guarantee students encounter only non-pathogenic varieties. Accidental isolation appears lower risk since laborious process requires particular growth media and techniques, but not a guarantee of safety as passage in an animal host to become pathogenic (Dragon and Rennie, 1995, 2001; Cieslak and Eitzen, 1999; Saile and Koehler, 2006).	Safety training begins with students to consider risks of isolating a pure culture. Staff training is also needed if potential contact. These events support following the guidelines for the special environment teaching lab resource (Woolverton and Woolverton, 2017). The risks from two different species vary but with educator concerns for student and staff safety, a risk assessment would rule out these sources from the environment for isolation.
***Dramatic examples persuading change***		
Guideline illustration “Do not subculture unknown microbes isolated from the environment because they may be organisms that require BSL2 practices and facilities” (Emmert, 2013). Faculty resist change citing outreach resembles traditional settings. Pasteur and Koch had risks as they searched for the cause of disease in their laboratories, kitchens, or the back room of a house. Faculty cite tradition having long taught “isolation of unknowns” as students swabbed from various sources: spices, soil, bathroom sinks, and toilets, or their own computers, cell phones, and hands. Potential pathogens are commonly found: *Salmonella* on sprouts, toxin-producing *Escherichia coli* on hamburger or water in the common coliform lab exercise, *Staphylococcus aureus* on skin or nasal passages, some with MRSA drug resistance. Online microbiology faculty believe students practicing aseptic technique at home safely. Risk and ethical dilemmas increase with human body source rectal and throat swabs, or animal sources from farm or pets. Faculty believe their farm animal sources to be free of disease. Clinical faculty believe their patient isolates to be safely contained. Each outbreak publicly raises awareness of emerging pathogens from animal sources and points to changing paradigms of outbreak from SARS, MERS, and COVID-19 (Salata et al., 2019; Guarner, 2020).	Wine, beer, and dairy simultaneously studied with causes of outbreaks (Blevins and Bronze, 2010). Overly dramatic and timely example provides a valid warning: - Virus causing COVID-19 with its probable animal origin has other possible domesticated drivers promoting its spread (Saegerman et al., 2020). - Faculty imagine having an outbreak originate from a swabbed isolate through zoonosis from a student’s pet (Halsby et al., 2014). - Multistate *Salmonella* outbreaks originating from pet hedgehogs (Anderson et al., 2017) - Ethical discussions of benefits vs. risks in artwork permeated with pathogenic bacteria (Fawcett and Dumitriu, 2018) - Nationwide outbreaks from United States microbiology teaching laboratories transported by cell phones traced back by federal investigations even from well-equipped laboratories within established laboratory safety practices.^2^,^3^,^4^	First step is prevention of hazards in the environment (Gostin and Wiley, 2016). As risks are identified, then faculty more open to change if a balance is found and the imposed mandates protect the public but with fairness and fairly distributed resources. With cost-effective, evidence-based promotion of health, then even if mandates are paternalistic but provide adaptable safe curricula, then all stakeholders could benefit.
***Alternate methods for environmental isolation***		
The 2019 Guideline addendum^5^ still warns against culturing from the environment; if done, then sealing and not further sampling or handling once microbes have been purified and propagated (Byrd et al., 2019). With absolutely no supplies (sometimes because shipments do not arrive), faculty create a microbiology lab but always seeking new safe methods that achieve learning outcomes. They try to fit the guidelines. Grow microbes on potatoes “sterilized” in a microwaved dish and inoculated with “sterilized” Q-tips and observe fungal growth on potatoes sealed to prevent exposure without opening or disturbing. Koch’s postulates are modeled with milk and yogurt cultures. Baker’s yeast shows size comparisons or microscopic eukaryotes with smaller prokaryotes and demonstrates a positive gram stain alongside fermenting bacteria in yogurt or sourdough.	Low-resourced teaching choices are made to find the balance between the need for the content in science education to promote scientific literacy or new discovery in inquiry-based learning with what can be done safely. One argument is traditional microbiology laboratory exercises isolating from environmental, their own human body, or other animal sources provide ease of source materials and low cost when it is not possible to order and maintain stock cultures. Without accessible alternate methods, the updated guidelines pose risks, and ethical decisions with updated guidelines must be made. Continued need for developed and shared curricula, free public health workshops, and recommendations for early reporting if anything amiss.	Update curricula: addressing cell phones, not taking laboratory notebooks home in backpacks, training for donning PPE, isolation, and social distancing. Determine alternate methods to properly prepare and view specimens for examination using microscopy, use pure culture and selective techniques to enrich for and isolate microorganisms and methods to identify microorganisms. Risk is mitigated if students do not open the plates once obtained, or if pure cultures are obtained by isolating from safer, non-pathogenic sources such as yogurt. If selective media is not available, obtain unused, expired plates from hospital clinical laboratory.•Use of alternative sliced potatoes lack firm colony formation, but useful for growth; gelatin lacks higher temperature incubation, but useful for aseptic technique; colored photographs and visual libraries useful for demonstrations and critical thinking exercises but lack hands on.

In response, an American Society for Microbiology (ASM) task force drafted and revised guidelines (Emmert, 2013; Woolverton, 2013; Byrd et al., 2019). An updated addendum,^5^ clarifies use of risk group RG1 organisms, and better accounts for the range of emergent issues in teaching facilities and laboratory practices. Guidelines are recommended, but not mandated. However, as enhanced safety guidelines evolve, they do not fully account for additional burdens that arise in under-resourced institutions. Assumptions of how microbiology is supposed to function often fail to include alternate viewpoints and practices in under-resourced settings.

Guidelines are assumed to be beneficial. Beneficence promotes a safety-ethics culture to prevent hazards, near-misses, or unreported incidents regardless of the science, size of laboratory or setting (Hill, 2016). Even small hazards in a teaching laboratory with untrained, introductory-level students may pose risk for undocumented laboratory-acquired infection (LAIs) (Carlberg and Yeaman, 2006). Harding and Byers (2006) review the epidemiological approach of distribution in populations and LAIs from research, clinical, and teaching. Outbreaks from teaching laboratories are low, but not systematically monitored or reported. Impacts include host susceptibility, behavioral factors, and the environment. Despite benefits, guidelines can also cause harm. Maleficence can occur when they are misunderstood, ill-fitting for the environment, or mandates produce unintended consequences.

Real-world biomedical challenges and public health ethical dilemmas are not new for under-resourced institutions with faculty struggling to provide microbiology laboratory courses safely. What appears non-standard for the mainstream is standard in another, termed “under-resourced” or “outreach” for the purpose of this article includes formal learning in different modalities: distance education, online and hybrid courses using do-it-yourself (DIY) at-home kits, citizen science, and laboratory courses hosted at different sites *via* a traveling lab bus. Assumptions begin with faculty having solid foundational understanding and respect for microorganisms and safety. Recognizing “one-size-fits-all” standards are not feasible, faculty and institutions adhere to a “good-faith effort” (Woolverton and Woolverton, 2017). However, what happens if these assumptions are not met, when a sole safety-trained scientist is alone pushing for reform, or when the upper administration is more concerned about the financial bottom line and appearance of effort without the true fidelity and commitment? These questions of safety and social justice in education are best addressed applying a public health ethics equity approach.

Under-resourced outreach teaching example University X provides a real-world scenario (Table 1). Challenges and failure to meet laboratory safety guidelines and other dilemmas are examined using a novel approach applying public health ethical analysis. Social justice issues surrounding the development and implementation of guidelines raises potential harm if mandated too harshly or when under-resourced institutions fail to respond well. Public health policymaking applies several frameworks. Schröder-Bäck et al. (2014) outlines seven principles to explore cases such as under-resourced University X: non-maleficence, beneficence, health maximization, efficiency, respect for autonomy, justice, and proportionality. Here, to assure that biosafety restrictions do not limit learning science in an unjust manner, this analysis raises awareness of the minority voice of under-resourced institutions.

## Non-Maleficence in Biosafety Guideline Compliance and Acceptable Risk in Changing Paradigms

Changing paradigms increase conflict by altering what constitutes acceptable risk, biosafety measures, and abilities to comply. The basis of bioethics and public health ethics is the Hippocratic oath “*primum nil nocere*,” taken as the first principle of non-maleficence and “do not harm.” There is a duty to educate as well as protect health. Lack of compliance to safety policy guidelines can harm, as can mandating too harshly. The framework of Kass (2001) asks the overall public health goals, program (guidelines) effectiveness, and potential burdens. If the goals, such as safety, cannot be implemented fairly to mitigate burdens, then we as a society collectively decide procedurally what should and should not be done to protect the health and maintain the education of communities. Bernheim et al. (2009) promotes procedural justice through ethical reflection of all affected groups being part of the decision-making process. The ASM community endeavors to duly discuss biosafety in educational teaching laboratories. Publication in journals requires explanation of adherence to safety guidelines; however, even this rigor can exclude. Burdens faculty face creates tensions when facing the moral code that they “ought” to meet guidelines – particularly when the institution fails them. When more voices are heard, then a balance for the under-resourced can procedurally be sought.

The framework of Baum et al. (2007, 2009) helps manage tensions in daily work by asking how the program guidelines advance wellbeing and respond to the needs of the community. Resolution of conflicts is determined by how burdens created by mandates (or even recommended guidelines) can be minimized through improved alternative approaches for fairness in equity and wellbeing. Rather than theoretically assuming safe practices, feasibility is considered in the daily practice.

The safety guidelines suggest risk assessment to prevent harm, e.g., student mishap, exposure, or a bigger contagion. The principle of non-maleficence is balanced with degrees of harm that would give the greater benefits. Guidelines are acceptable when other harms they create are limited. This utility by Bentham’s measure of wellbeing evokes the doctrine of utilitarianism of providing the greatest good for the greatest number (Bentham, 1781, 1996). However, utilitarianism is flawed since consequences are not predictable. In applying consequentialist theory, the actions of utility deemed most correct is one that provides the most benefit gain for the majority (Roberts and Reich, 2002). Utilitarianism is challenged by social justice and the needs of the minority if utility is only increased for the majority.

When all voices are not heard equally, policy guidelines can result in harmful unintended consequences. Harm results if fear or administrative ignorance results in course cancellations. Without alternative approaches, under-resourced educators face burdens of teaching laboratory courses with inadequate safety vs. offering no course at all.

University X (Table 1), dedicated to low tuition for lower socioeconomic (SES) students, limits funding resources, temporarily hires faculty, lacks lab manual curricula, and lacks institutional safety officers. Overwhelmed by deficient support, faculty rely on piecemeal lab kits “on hand” and think outside the box to teach authentic science, often putting faculty in precarious positions with egregious practices to get the job done. The slippery slope begins when faculty trying to encourage greater student engagement use “let’s give it a shot” attitude and “let’s try it and see” to justify their choices (Tippins et al., 1993). Educators knowledgeable of the risks advocate for support, sometimes as the lone voice seeking institutional change. The unguided administration can balk and retaliate resulting in microbiology laboratory course cancellations and in doing so, denying access to education and science literacy. Ethical frameworks applied by the scientific community can help address the underlying moral conflict of stringent biosafety guidelines causing harm.

## Beneficence, Health Maximization, and Efficiency

The crux lies in balancing acceptable “tradeoffs” between non-maleficence degrees of harm and the second principle of beneficence, the obligation to produce benefit. To weigh the beneficence of guidelines, risks are ascertained with the broader view of the third principle, health maximization including the greater population. Risk assessment of small-scale threats are similar to the larger scale National Response Framework emergency management cycle: prevention of hazards, risk identified, and fairness imposing mandates to protect (Gostin, 2000a,b,c, 2010; Gostin and Powers, 2006; Gostin and Wiley, 2016). Public health law ties mandates to different degrees of enforceable governmental regulation and even non-mandated, non-enforced guidelines imply obligation through semantics (Harmon, 2016). The moral burden put upon an instructor, whether sufficiently supported or not, and the guilt and culpability that would be incurred if an accident occurred increases the ethical dilemma.

Guidelines elicit a public health benefit to student populations. If pressure from restrictive measures threatens the course loss, then a counter benefit is for the greater public good with an obligation to provide laboratory education supporting science literacy and the welfare of others. Science literacy and microbial appreciation are increasingly important at every level of our global society to understand how scientific understanding changes through evidence. We need laboratory courses for a full science curriculum for our future scientists and health care workers, as well as policy makers, agencies, and general population (Timmis et al., 2019). In sustaining more science courses, then the fourth principle, efficiency, promotes greater impacts. By assuring evidence-based, cost-effective, safe practices for under-resourced education, then science literacy is maintained without short-changing learning even with subpar institutional support.

These trade-offs are exemplified as University X struggles with more stringent guidelines when applying skills of the standard “isolation of unknown” as one form of discovery meeting ASM curricular learning outcomes.^6^ Outside of the standard of practice, instructors still resort to isolation practices not consistent with guidelines. Lacking stock cultures, students swab different environmental, their own human body, or other animal sources to isolate unknown microorganisms. Microbes grow, students streak to isolate pure culture colonies, and stain to identify. Risk increases working with environmental cultures if a pathogen is propagated in pure culture; yet, reliance on these traditional practices provides ease of source materials and low cost when it is difficult to order and maintain stock cultures. Alternate methods are sought within guideline recommendations (Table 1). Course-based undergraduate research experiences (CURES) for institutions that desire applications of real-world, authentic research experiences but may lack research infrastructure have additional needs (Alkaher and Dolan, 2014; Auchincloss et al., 2014; Davis et al., 2017). Some have expanded Tiny Earth soil projects for broader educational applications with adapted protocols for genomic identification and pivot to online with the pandemic^7^ (Basalla et al., 2020).

For new educators, even well trained from R1 research institutions, temporary visiting professor, or adjuncts, the shift to low-resourced education can be daunting with subpar institutional support, proving difficult to navigate and ensure safe, meaningful curricula. Institutions of any type might struggle when modalities change due to life-altering pandemics. When courses globally shift online, many instructors face new challenges teaching laboratory courses authentically and safely, but more so if institutional resources and instructor preparedness are limited (Hodges et al., 2020; Procko et al., 2020; Rapanta et al., 2020). Even providing critical thinking curricula can be a challenge when resources are limited (Aparna et al., 2020, this issue; Song et al., 2016). Hierarchy designates safety officers bear the burden of liability, but places a higher burden of culpability on the instructor. Frustration mounts when the institution lacks a safety officer and inability to publish with embarrassing institutional breaches.

When instructor practices are noncompliant with guidelines, they may need deeper investigation to determine risks of students potentially isolating a pathogenic microorganism from environmental sources (Table 1). Even the easily adapted “handwashing” or “disinfectant” labs with resistant bacteria found in soil and paper towels are not without risk since any immunocompromised situation, even pregnancy, increases risks. Although most human infectious disease pandemics originate from cross-species transmission, these are rare in a teaching laboratory (Hughes et al., 2010). However, several dramatic and timely examples provide a valid warning (Table 1). Any anomaly away from “normal science” pushes the paradigm change.

## Respect for Autonomy vs. Top-Down “Paternalistic” Mandates

Within public health frameworks, paternalistic guidelines mandated from the top-down are contrasted with the fifth principle of respect for autonomy as a moral consideration (Childress et al., 2002). Academic decision-making by institutions and instructors to comply with guidelines, or not, is a moral choice if guidelines limit individual liberties, or academic freedom. A disadvantage to autonomy is the ethical burden of poor compliance: the student choosing not to comply, the instructor desiring autonomy in teaching strategies, or the administration failing to adequately provide support.

Some educators find themselves advocating for policy changes at their own institutions but in precarious positions of power dynamics. If educators advocate too firmly or take a whistleblowing approach, then courses could be canceled and jobs lost. University X with an inability to comply (or administrative choice not to allocate funds) may result in a knee jerk reaction to cancel microbiology courses putting future generations at risk with the increasing fear of science and lack of knowledge that protects us all and limits justice.

## Justice for Equitable Access

Baylis et al. (2008) highlight frameworks that focus on a social justice approach for the common good. This calls upon relational autonomy, solidarity of common interests rather than “us and them,” and justice in the fairness of how decisions are made. With the consequentialist approach, respect for individual stakeholder interests is unbalanced. Taking the deontological, duty-based approach, policies holding social justice take priority for the most good. Supporting faculty in being able to adhere to a duty-based approach applies normative ethical theory; a moral code determines if an action is right or wrong under a set of rules (Bellefleur and Keeling, 2016). However, if the rules only assume adequate resources, then this adds burden to the duty under-resourced educator’s bear.

When the need to follow updated safety guidelines poses threats to course cancellations, then the under-resourced institutions are at further risk. To increase social justice, education needs to reach beyond those in college who cannot afford education by expanding the greater good through promoting science literacy. A hidden part of the unintended consequences of this dilemma is that more of those who come from lower SES attend these under-resourced colleges (Engberg and Allen, 2011). Engaged learning such as laboratory courses offered at community colleges, minority-serving institutions, and from educational opportunities provided through the United States military contracts at home and abroad along with other outreach settings is valuable. Engagement matters in student retention and success (Kuh and Pascarella, 2004; Pike, 2004; Kuh et al., 2006), so this potential cancellation of courses presents a social justice dilemma by limiting science courses that keep low SES students on the trajectory toward graduation, further degree completion, and next steps. Despite increasing college enrollments for underrepresented ethnic minorities, the trends for educational attainment of science, technology, engineering, and mathematics (STEM) degrees and overall graduation within 6 years show disparities (de Brey et al., 2019; McFarland et al., 2019; Cahalan et al., 2020). It is fundamental that resources are attainable, guidelines are equitable, and stigma is limited.

Microbiology fluency, laboratory practices, and equitable access to these skills must be met for mastery of concepts through equitable opportunities and completion (National Academies Science Engineering Medicine, 2018). The vision that all students who desire access to learning, should obtain it is addressed through the Partnership for Undergraduate Life Sciences Education (PULSE) with rubrics to measure and promote Vision and Change (Brancaccio-Taras et al., 2016). Although this is useful to achieve goals of modern competencies (Woodin et al., 2010) across different institution types, some under-resourced institutions such as University X are missed in this revolution and feel the gap.

## Conclusion with Proportionality of Individual Freedom with Public Good

By applying public health frameworks, the primary goal is to mitigate harm in populations: harm from risk to health and from the unintended consequences of policies. The seventh and last principle of proportionality balances that the probable benefits of guidelines for the public good outweigh the infringement on the few. While considering non-maleficence, then compliance to guidelines could promote equitable safety, but harm could occur if equitable educational opportunity is lost. Guidelines with implied instructor culpability, or mandated with severe restrictions and without solutions, create inequitable gaps.

A solution of least infringement and equity to ensure stringent guidelines do not compromise student learning is to provide specifically written safe curricula to aid compliance. There are alternate methods to achieve learning outcomes and still promote compliance (Table 1). When advocating risk assessment, guidelines specifically recommend ideas to address the challenges under-resourced faculty face. This is attempted through contributions from diverse institutional types compiling creative ideas for non-traditional settings in open-source-shared curricula, i.e., ASM’s Microbe Library^8^ or Course Source.^9^ Broader dissemination to institutions is a proposed solution if educators themselves lack knowledge. This practice of under-resourced shared teaching ideas helps mitigate harm of excess burden placed on the instructor to meet guidelines when lacking institutional support. Reaching out through the community using an evaluated process with confidentiality assures all legitimate stakeholder voices are involved in providing equitable opportunities and protection for those from underserved populations most at risk of under-resourced courses. In this manner, justice would distribute an equitable, compliant, curriculum, while burdening all to comply.

Educators collectively assure healthy conditions in microbiology teaching laboratory courses philosophically through normative ethics: educators “ought” to be informed by updated guidelines, “ought” not to continue methods with higher risk, and institutions “ought” to provide faculty the curricular and biosafety officer support needed for optimal safety within constraints. Although utilitarianism allows protection and greater accessibility, we must still rely on morality as defined by social contract theorists to apply social justice frameworks for the underserved. Sometimes individual observation when seeing something amiss begs a moral duty to make the correction. Rather than waiting for adverse events, some try whistleblowing in the case of non-compliance or institutional protection. A notable voice, Dr. Li, first signaling the COVID-19 outbreak stated “I think a healthy society should not have just one voice” (Green, 2020). It is for this reason that the voices of the under-resourced must be heard in providing solutions.

## Author Contributions

The author confirms being the sole contributor of this work and has approved it for publication.

### Conflict of Interest

The author declares that the research was conducted in the absence of any commercial or financial relationships that could be construed as a potential conflict of interest.

## Footnotes

^1^https://www.cdc.gov/training/QuickLearns/biosafety/

^2^https://www.cdc.gov/salmonella/2011/lab-exposure-1-17-2012.html

^3^https://www.cdc.gov/salmonella/typhimurium-labs-06-14/index.html

^4^https://www.cdc.gov/salmonella/typhimurium-07-17/index.html

^5^https://asm.org/Guideline/ASM-Guidelines-for-Biosafety-in-Teaching-Laborator

^6^https://asm.org/Guideline/ASM-Curriculum-Guidelines-for-Undergraduate-Microb

^7^https://tinyearth.wisc.edu/

^8^https://www.asmscience.org/VisualLibrary

^9^https://www.coursesource.org/
